# Pediatric Case of Li–Fraumeni Syndrome in Honduras

**DOI:** 10.1155/2021/6612802

**Published:** 2021-01-11

**Authors:** R. Martínez-Beckerat, C. Alas-Pineda, M. Melgar-Gonzales, B. Mejía-Raudales, N. Andino-Paz, S. Bejarano-Cáceres, J. Chiang

**Affiliations:** ^1^Hospital Nacional Dr. Mario Catarino Rivas, San Pedro Sula, Cortés 21102, Honduras; ^2^Facultad de Medicina y Cirugía, Universidad Católica de Honduras-Campus San Pedro y San Pablo, San Pedro Sula, Cortés 21102, Honduras; ^3^Universidad Nacional Autónoma Honduras en el Valle de Sula, Escuela Universitaria de las Ciencias de la Salud, San Pedro Sula 21102, Honduras; ^4^Liga Contra el Cáncer de Honduras, San Pedro Sula, Cortés 21104, Honduras; ^5^Department of Pathology, St. Jude Children's Research Hospital, Memphis, TN 38105, USA

## Abstract

Li–Fraumeni syndrome is an inherited, autosomal dominant disease. It is categorized as a rare disease caused by mutations of the *TP53* gene, which causes increased susceptibility of the patients and their children to many types of cancer. Choroid plexus tumor is rare, which occurs in 0.3 cases per 1,000,000 people, of which 40% turn out to be carcinomas. We present a 12-year-old boy with a history of worsening headaches of more than one month, gait disturbance, projectile vomiting, and right hemiparesis. An intraventricular tumor was identified in the occipital of the left lateral ventricle, which turned out to be a TP53-mutant choroidal plexus carcinoma.

## 1. Introduction

Li–Fraumeni syndrome (LFS) was first reported in 1969 by Frederick Li and Joseph Fraumeni; hence, the name of the syndrome [1]. LFS, also known as sarcoma, breast, leukemia, and adrenal gland (SBLA) syndrome, is a rare autosomal dominant hereditary disorder caused by mutations in different levels of TP53 germlines, a tumor suppressor gene that regulates the cell cycle. In some cases, the mutations can occur de novo, not from hereditary, and in 70%, they are due to mutations in *TP53* [2–4].

In this syndrome, the literature is not enough to estimate a definite and universal prevalence. However, a recent study placed the prevalence of pathogenic and likely pathogenic germline TP53 variants in 1 : 3,555 to 1 : 5,476 on the general population [3]. Furthermore, advances in genomics report that LFS has 70% of genetic penetrance in males and 90% of that in females. This shows us a syndrome that requires careful monitoring of the family lineage [5, 6].

Epidemiological studies reveal that most patients with LFS develop primary cancer at a young age. The most prominent types of cancer associated with LFS are sarcomas, brain tumors, breast cancer, leukemia, and adrenal carcinoma. Other types of cancers associated with LFS include melanoma, lymphoma, renal tumors, laryngeal cancer, lung cancer, colon cancer, ovarian cancer, testicular cancer, and thyroid cancer [4, 6].

## 2. Clinical Case

The patient is a 12-year-old male from a rural area of Honduras who was evaluated by pediatric emergency services at a second-level hospital of Honduras for a moderate headache with 1 month of persistence. At the time of evaluation, the patient referred the headache to be pulsatile and holocranial, with a duration of several hours, and is unresponsive to NSAIDs. Additionally, the patient presented with episodes of projectile vomiting, seizures, right hemiparesis, and progressive gait disturbance. No relevant pathological history is noted.

**Figure 1:**
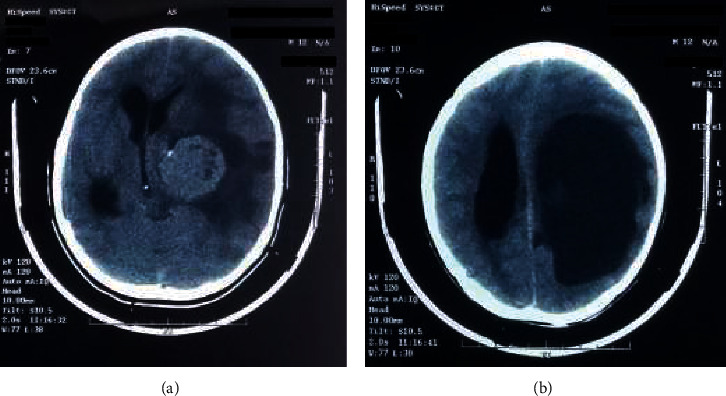
Preoperative simple CT of the brain. (a) 10 × 7 cm mass in the posterior horn of the ventricle. (b) Lesion at the left parietal region accompanied by secondary ventricular dilation.

During physical examination, the patient remained in a postictal state with neurological deterioration, memory impairment, and conduct disorder. There were also signs of intracranial hypertension. A CT was performed upon admission, which revealed a cystic lesion with irregular borders in the left parietal region that displaced the midline. Perilesional edema was also observed, as well as hydrocephalus (Figure 1). The lesion was surgically excised, revealing a 10 × 7 cm mass taken from the posterior horn of the lateral ventricle. An external ventricle shunt was put in place to maintain intracranial pressure. There were no intraoperative or postoperative complications.

**Figure 2:**
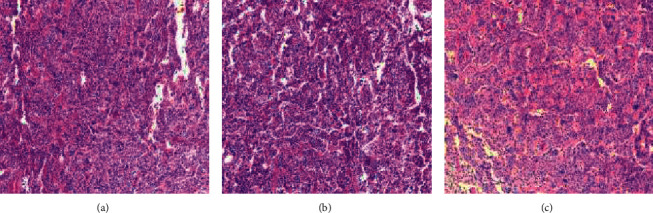
Biopsy analysis at 50%. The histologic section showing fragments of a hypercellular neoplasm with mixed solid and papillary architecture. The tumor cells demonstrate moderate to marked nuclear pleomorphism. Scattered mitotic activity is noted.

Histopathological studies revealed a focal papillary neoplasia consistent with choroid plexus carcinoma (grade III). Due to the association of choroid plexus carcinomas with autosomal dominant germline mutations of the *TP53* gene, the patient's three-generation family history was reviewed. In the paternal side, multiple cases of liver and cervical cancers were discovered. The maternal side did not present a neoplastic history. Confirmatory pathological and immunohistochemical studies were performed at Saint Jude Children's Research Hospital in Memphis, Tennessee, USA. The study reported the finding of a hypercellular neoplasia with pleomorphic tumors cells, mixed patterns of papillary and solid growth, and scattered mitotic activity. Additionally, immunohistochemical studies were positive for cytokine CAM 5.2 and p53, which strongly marked the nuclei of the tumor cells (Figure 2). These studies confirmed the presence of a *TP53*-mutant choroid plexus carcinoma.

**Figure 3:**
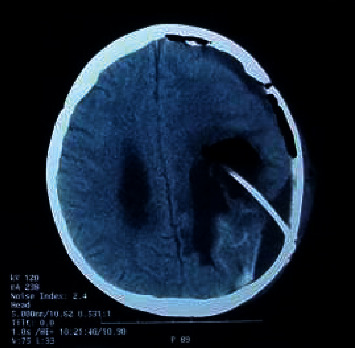
Postoperative simple CT of the brain.

The patient had a satisfactory course after surgical resection. Oncological follow-ups were carried out, with six cycles of chemotherapy (with ifosfamide, carboplatin, and etoposide). CT scan after the surgery revealed a subdural collection of approximately 134 ccs in the right frontotemporoparietal region (Figure 3). No remaining mass was observed, and the lateral ventricles were of standard size and shape. According to the latest follow-up, the patient is asymptomatic, although he does refer to some loss of strength in the right side.

## 3. Discussion

*TP53* is a vital gene because it maintains homeostasis at a molecular level, regulating the cell cycle through the encoding of the p53 protein, known as the “guardian of the genome” [7, 8]. Therefore, it is not surprising that any mutation in the *TP53* gene would substantially impact the performance of cells and give rise to a variety of pathologies. A total of 200 polymorphisms associated with this gene have been identified today [7].

Although Li's and Fraumeni's first study began with 4 patients in 1969, by 1988, information of 24 different families was involved in the studies; 151 members of those families were diagnosed with some form of cancer. Because of this, a predictive pattern was observed and analyzed. The majority of these subjects had developed five types of cancer: sarcomas, breast cancer, leukemia, adrenal carcinoma, and brain tumors. Of the latter, a less common type was choroid plexus carcinoma. According to Tena-Suck et al., choroid plexus carcinoma represents only 0.3–0.6% of the tumors associated with the *TP53* gene mutation [9]. According to this study and a similar study from St. Jude Children's Hospital, patients under the age of 15 represent 2–4% of those diagnosed with choroid plexus carcinoma [10–12].

The annual incidence of choroid plexus carcinoma is 0.3/1,000,000 [10]. The WHO classifies choroid plexus tumors as grade I, choroid plexus papilloma; grade II, atypical choroid plexus papilloma; and grade III, choroid plexus carcinomas [13]. Choroid plexus papillomas represent 57.8% of all cases, while atypical choroid plexus papillomas represent 1.8% and choroid plexus carcinomas, 40% [11].

Several studies suggest that the rare appearance of choroid plexus tumors is associated with the TP53 gene mutations [14–16]. In this case, the patient had the tumor in the lateral ventricle, a region affected in 54% of patients with choroid plexus tumors. It is the most affected region because only 11% affects the third ventricle and 8%, the cerebellopontine angle [17].

Characteristic signs and symptoms (headache, hydrocephalus, gait disturbances, and projective vomiting) are indicative of plexus choroid tumors, but not pathognomonic. The diagnosis is based on histopathological findings, with surgical excision playing an important role. As for the treatment of choroid plexus tumors, there is little evidence that fully supports a specific protocol. Tumor resection seems to be essential; however, only 30% of these tumors can be resected completely [18].

## 4. Conclusion

LFS is a rare pathology with high penetrance within family members that possess the *TP53* gene mutation. Patients with LFS are at a higher risk of developing different types of cancer, namely, sarcomas, breast cancer, leukemia, adrenal carcinomas, and brain tumors. The definite diagnosis is made with histopathological findings, particularly in the case of choroid plexus tumors. These tumors commonly affect younger patients, like the patient from this case. Treatment of choroid plexus tumors continues to be surgical resection, with adjuvant chemotherapy.

## Data Availability

Patient file is stored at the statistics department of Hospital Mario Catarino Rivas.
